# A Functional Polymorphism in the 3'-UTR of PXR Interacts with Smoking to Increase Lung Cancer Risk in Southern and Eastern Chinese Smoker

**DOI:** 10.3390/ijms151017457

**Published:** 2014-09-29

**Authors:** Lisha Zhang, Fuman Qiu, Xiaoxiao Lu, Yinyan Li, Wenxiang Fang, Lan Zhang, Yifeng Zhou, Lei Yang, Jiachun Lu

**Affiliations:** 1 The State Key Lab of Respiratory Disease, the Institute for Chemical Carcinogenesis, Collaborative Innovation Center for Environmental Toxicity, Guangzhou Medical University, 195 Dongfengxi Road, Guangzhou 510182, China; E-Mails: lishazhang@126.com (Li.Z.); fuman009@163.com (F.Q.); yyli68@hotmail.com (Y.L.); Wenxiangfang86@126.com (W.F.); zhanglan_gzhmu@163.com (La.Z.); 2 School of Arts and Sciences, Colby-Sawyer College, New London, NH 03257, USA; E-Mail: xiaoxiao.lu@my.colby-sawyer.edu; 3 Department of Genetics, Collaborative Innovation Center for Environmental Toxicity, Medical College of Soochow University, 199 Renai Road, Suzhou 215123, China; E-Mail: zhouyifeng@suda.edu.cn

**Keywords:** pregnane X receptor (PXR), lung cancer, susceptibility

## Abstract

Pregnane X receptor (PXR) is an important member of the nuclear receptor superfamily that copes with various endobiotic and xenobiotic stimuli, such as carcinogens by regulating an array of environmental response genes. Low *PXR* expression has been shown to promote tumor initiation and metastasis. The aim of the current study was to investigate whether the single nucleotide polymorphisms (SNPs) of *PXR* could alter lung cancer susceptibility in Chinese by affecting the function or expression of *PXR*. We genotyped three putatively functional SNPs of *PXR* (*i.e.*, rs3814055C>T, rs3732360C>T, and rs3814058C>T) and analyzed their associations with lung cancer risk in a two-stage case-control study with a total of 1559 lung cancer cases and 1679 controls in the southern and eastern Chinese population. We found that in comparison to the rs3814058CC common genotype, the rs3814058T variants (TC/TT) which is located in the 3'-untranslated region (3'-UTR) of *PXR* conferred a consistently increased risk of lung cancer in both the southern Chinese (odd ratios (OR) = 1.24, 95% confidence interval (CI) = 1.03−1.49) and the eastern Chinese (OR = 1.33, 95% CI = 1.02−1.75). The variants also significantly interacted with smoking on increasing cancer risk (*p* = 0.023). Moreover, lung cancer tissues with the rs3814058T variants showed significantly lower *PXR* expression than those with rs3814058CC genotype in the smokers (*p* = 0.041). These results suggested that the rs3814058C>T polymorphism of *PXR* interacts with smoking on increasing lung cancer risk in Chinese smokers, which might be a functional genetic biomarker for lung cancer.

## 1. Introduction

Lung cancer is the most common type of malignant tumor and the leading cause of cancer-related death both in China and worldwide. Data from the population-based national central cancer registry of China showed that 605,946 new cases and 486,555 deaths of lung cancer had occurred in 2010 [1]. Meanwhile, the number is on the increase and will cause heavy social burdens with respect to the large number of smokers in China. Tobacco smoking is the major risk factor of lung cancer [2]. Tobacco contains several well-established chemical carcinogens, such as polycyclic aromatic hydrocarbons (PAHs), tobacco-specific nitrosamines, and metals [3]. These carcinogens can bind to DNA and form DNA-adducts, which can further damage DNA if not repaired and thus induce genomic instability as well as mutations in some cancer-related genes. Both would contribute to the susceptibility and development of human cancers [4,5]. In the process, the body’s adaptive defense system can help the body resist damage through detoxification and excretion of these carcinogens by regulating the absorption and metabolism of these carcinogens. Interestingly, several single nucleotide polymorphisms (SNPs) in molecules of the body’s adaptive defense system have been reported to be associated with risk of various human diseases including cancer [6,7,8,9].

The nuclear pregnane X receptor (PXR, also named NR1I2) is an important component of the body’s adaptive defense system against toxic xenobiotics and endogenous metabolites. PXR belongs to the nuclear receptor superfamily of ligand‑regulated transcription factors and regulates the transcription of numerous metabolic enzymes implicated in cellular response to xenobiotics [10,11]. PXR was originally shown to serve as a master transcriptional regulator of xenobiotic-inducible *cytochrome P450* (*CYP*) genes [12,13], and then was found to play a role in the activation of *CYP3A4* and *CYP2B6*, all of which belong to a gene family that plays pivotal roles in metabolic transcription of tobacco-derived carcinogens, especially PAHs, and development of lung cancer [14,15,16,17]. If not be removed by the CYPs, metabolic intermediates of carcinogens are often highly active and can induce initiation and promotion of tumor [18,19].The *PXR* expression was associated with the inducibility of *CYP* genes’ activities in lung, suggesting that PXR participates in the inactivation of tobacco-carcinogenic agents and may be involve in the development of lung cancer [20]. Disordered *PXR* expression has been reported in a variety of tumor types, correlating not only with drug resistance but also with the proliferation, apoptosis, and prognosis of cancer [21,22,23,24]. Low *PXR* expression was also observed in lung cancer cells [25].

The human *PXR* gene is located on human 3q12-13.3, coding the PXR protein that contains a *N*-terminal domain, a DNA-binding domain (DBD) and a *C*-terminal ligand-binding domain (LBD) [11,26]. Recently, several studies have reported that SNPs of *PXR* were associated with risk of several diseases, including Barrett’s esophagus (BE), nonalcoholic fatty liver disease and inflammatory bowel disease [6,8,9]. However, no study has yet tested the associations between SNPs of *PXR* and lung cancer risk. It is well known that SNPs that are located in the promoter, 5'-untranslated region (5'-UTR), exons, and 3'-UTR of gene sequences may influence the expression or the structure of genes and thus supported to be putatively functional [20,25].The putatively functional SNPs of *PXR* may affect the expression or function of *PXR*. On account of the fact that PXR may play roles in lung carcinogenesis, we hypothesized that these putatively functional SNPs in the *PXR* gene were associated with lung cancer susceptibility in Chinese.

In two independent case-control studies, we firstly genotyped three putatively functional SNPs of* PXR* (*i.e.*, rs3814055C>T in the 5'-untranslated region (5'-UTR); rs3732360C>T and rs3814058C>T in the 3'-untranslated region (3'-UTR)) with common frequency (*i.e.*, minor allele frequency in Chinese > 5%) in a southern Chinese population with a total of 1056 cases of lung cancer patients and 1056 sex and age frequency-matched controls, and analyzed the associations between the SNPs and lung cancer risk; we then validated the promising association in an eastern Chinese population. Quantitative reverse transcription PCR (qRT-PCR) was further performed to assess the genotype-phenotype correlation between the promising SNP and *PXR* mRNA levels in lung cancer tissues.

## 2. Results

### 2.1. PXR Genotypes and Lung Cancer Risk

As shown in Table 1, all observed genotype frequencies of *PXR* SNPs were in agreement with the Hardy-Weinberg equilibrium in the cancer-free controls (*p* > 0.05 for all). In the discovery set of the southern Chinese, we found that the SNP rs3814058C>T has a significant difference in frequency distributions of genotypes between the lung cancer cases and controls (*p* = 0.033). Compared with individuals carrying the common rs3814058CC genotype, those with the rs3814058T variants (TC or TT) had a significantly increased risk of lung cancer (odds ratio (OR) = 1.24; 95% confidence interval (CI) =1.03–1.49; *p* = 0.023). The results from the validation set of the eastern Chinese further confirmed the significant association above, that the carriers of rs3814058T variants had a significant increased cancer risk (OR =1.33, 95% CI = 1.02–1.75; *p* = 0.026). We then combined the two populations as a merged set to increase the study power. In the merged set of a total of 1559 lung cancer cases* versus* 1679 controls, the carriers of rs3814058T variants had 1.25-folds increased risk of lung cancer (OR =1.25, 95% CI = 1.08-1.45; *p* = 0.004) compared with the rs3814058CC genotype. However, because no significant association was observed between the two SNP rs3814055C>T (*p* = 0.836), rs3732360C>T (*p* = 0.758) and lung cancer risk in the southern Chinese, we did not validate their associations with lung cancer risk in the eastern Chinese. In addition, the frequency distributions of demographic characteristics of the discovery set and the validation set are shown in Table S1.

**Table 1:** Frequency distributions of genotypes of *pregnane X receptor* (*PXR*) single nucleotide polymorphisms (SNPs) and their associations with the risk of lung cancer.

Genotypes	Case *n* (%)	Control *^a ^n* (%)	*p* Value*^ b^*	Crude OR (95% CI)	Adjusted OR*^ c^* (95% CI)
Discovery set					
Total no. of subjects	1056	1056			
rs3814055C>T					
CC	693 (65.6)	706 (66.9)	0.836	1.00	1.00
TC	328 (31.1)	316 (29.9)		1.06 (0.88–1.27)	1.06 (0.88–1.28)
TT	35 (3.3)	34 (3.2)		1.05 (0.65–1.70)	1.05 (0.65–1.70)
rs3732360C>T					
CC	347 (32.9)	346 (32.8)	0.758	1.00	1.00
TC	520 (49.2)	533 (50.5)		0.97 (0.80–1.18)	0.97 (0.80–1.18)
TT	189 (17.9)	177 (16.7)		1.07 (0.83–1.37)	1.07 (0.83–1.37)
rs3814058C>T					
CC	315 (29.8)	365 (34.6)	**0.033**	1.00	1.00
TC	505 (47.8)	491 (46.5)		1.19 (0.98–1.45)	1.19 (0.98–1.45)
TT	236 (22.4)	200 (18.9)		1.37 (1.07–1.74)	**1.36 (1.07–1.73)**
TC + TT	741 (70.2)	691 (65.4)		1.24 (1.04–1.49)	**1.24 (1.03–1.49)**
Validation set					
Total no. of subjects	503	623			
rs3814058C>T					
CC	122 (24.2)	185 (29.7)	0.093	1.00	1.00
TC	254 (50.5)	303 (48.6)		1.27 (0.96–1.68)	1.28 (0.96–1.70)
TT	127 (25.3)	135 (21.7)		1.43 (1.02–1.99)	**1.47 (1.05–2.05)**
TC + TT	381 (75.8)	438 (70.3)		1.32 (1.01–1.72)	**1.33 (1.02–1.75)**
Merged set					
Total no. of subjects	1559	1679			
rs3814058C>T					
CC	437 (28.0)	550 (32.8)	**0.006**	1.00	1.00
TC	759 (48.7)	794 (47.2)		1.20 (1.03–1.41)	**1.20 (1.02–1.41)**
TT	363 (23.3)	335 (20.0)		1.36 (1.12–1.66)	**1.38 (1.13–1.67)**
Dominant model					
CC	437 (28.0)	550 (32.8)		1.00	1.00
TC + TT	1,122 (72.0)	1,129 (67.2)		1.25 (1.08–1.45)	**1.25 (1.08–1.45)**

*^a^* The observed genotype frequencies were all in agreement with the Hardy-Weinberg equilibrium (*p*^2^ + 2*pq* + *q*^2^ = 1) in the control subjects of all sets (*p* > 0.05 for all); *^b^* *p* value from the chi-square test to assess the differences in frequency distributions of genotypes of *PXR* SNPs between cases and controls; *^c^* Adjusted in a logistic regression model that included age, sex, smoking status, alcohol use, and family history of cancer; and Bold numbers mean that the difference or association was statistically significant.

### 2.2. Stratification Analysis

As shown in Table 2, there was a significant interaction (multiplication model: *p* = 0.023) between the SNP rs3814058C>T and smoking on the risk of lung cancer with the OR value equaling to 1.33 (95% CI = 1.02–1.73) in the stratum of current smokers and 1.92 (95% CI = 1.31–2.81) in former smokers while no significant effect observed (OR = 1.06, 95% CI = 0.85–1.31) in the stratum of non-smokers. Also, the rs3814058T variants significantly interacted with pack-years smoked on increasing lung cancer risk (*p* = 0.019). In addition, for other stratified factors, we did not find any significant differences in associations between rs3814058T variants and risk of lung cancer in each stratum (multiplicative interaction test: *p* > 0.05 for all).

**Table 2:** Stratification analysis of the *PXR* rs3814058C>T genotypes by selected variables in lung cancer patients and controls.

Variables	Cases (*n* = 1559)		Controls (*n* = 1679)		Adjusted OR *^a^* (95% CI)	*p* *^b^*
	TG + TT *n* (%)	CC *n* (%)	TG + TT *n* (%)	CC *n* (%)	TC + TT* vs.* CC	
Age (years)						
≤60	586 (72.4)	223 (27.6)	595 (67.8)	282 (32.2)	1.24 (1.01–1.54)	0.884
>60	536 (71.5)	214 (28.5)	534 (66.6)	268 (33.4)	1.24 (1.00–1.54)	
Sex						
Male	793 (72.7)	298 (27.3)	787(66.4)	398 (33.6)	1.35 (1.13–1.62)	0.107
Female	329 (70.3)	139 (29.7)	342(69.2)	152 (30.8)	1.03 (0.78–1.35)	
Smoking status						
Current	371 (72.4)	141 (27.6)	354 (66.5)	178 (33.5)	**1.33 (1.02–1.73)**	**0.023**
Former	241 (77.2)	71 (22.8)	150 (64.4)	83 (35.6)	**1.92 (1.31–2.81)**	
Never	510 (69.4)	225 (30.6)	625 (68.4)	289 (31.6)	1.06 (0.85–1.31)	
Pack-years smoked						
≥20	457 (73.2)	167 (26.8)	159 (33.2)	320 (66.8)	**1.34 (1.04–1.75)**	**0.019**
<20	155 (77.5)	45 (22.5)	119 (32.5)	247 (67.5)	**1.93 (1.28–2.93)**	
0	510 (69.4)	225 (30.6)	272 (32.6)	562 (67.4)	1.10 (0.89–1.36)	
Drinking status						
Ever	218 (74.4)	75 (25.6)	231 (67.5)	111 (32.5)	1.48 (1.03–2.11)	0.427
Never	904 (71.4)	362 (28.6)	898 (67.2)	439 (32.8)	1.21 (1.03–1.43)	
Family history of cancer						
Yes	91 (70.5)	38 (29.5)	98 (66.7)	49 (33.3)	1.15 (0.68–1.94)	0.821
No	1031 (72.1)	399 (27.9)	1031 (67.3)	501 (32.7)	1.25 (1.07–1.47)	
Family history of lung cancer						
Yes	40 (76.9)	12 (23.1)	28 (65.1)	15 (34.9)	1.82 (0.67–4.91)	0.082
No	1082 (71.8)	425 (28.2)	1101 (67.3)	535 (32.7)	1.23 (1.06–1.43)	
Histological types						
Adenocarcinoma	431 (70.1)	184 (29.9)	1129 (67.2)	550 (32.8)	1.14 (0.93–1.40)	
Squamous cell carcinoma	385 (73.1)	142 (26.9)			1.31 (1.05–1.63)	
Large cell carcinoma	45 (68.2)	21 (31.8)			1.07 (0.63–1.81)	
Small cell lung cancer	152 (78.8)	41 (21.2)			1.83 (1.28–2.63)	
Other carcinomas *^c^*	109 (69.0)	49 (31.0)			1.08 (0.76–1.54)	
Stages						
I	136 (68.0)	64 (32.0)	1129 (67.2)	550 (32.8)	1.04 (0.76–1.42)	
II	105 (71.4)	42 (28.6)			1.22 (0.84–1.77)	
III	357 (72.9)	133 (27.1)			1.31 (1.05–1.64)	
IV	524 (72.6)	198 (27.4)			1.30 (1.07–1.57)	

*^a^* ORs were adjusted for age, sex, smoking status, drinking status, family history of cancer in a logistic regression model; *^b^ p* value from the multiplicative interaction test between the rs3814058C>T genotypes and selected variables on cancer risk in a logistic regression model; and ***^c^*** Mixed-cell or undifferentiated carcinoma; and Bold numbers mean that the difference or association was statistically significant.

### 2.3. Association between the rs3814058C>T Genotypes and mRNA Levels of PXR Gene

**Figure 1:**
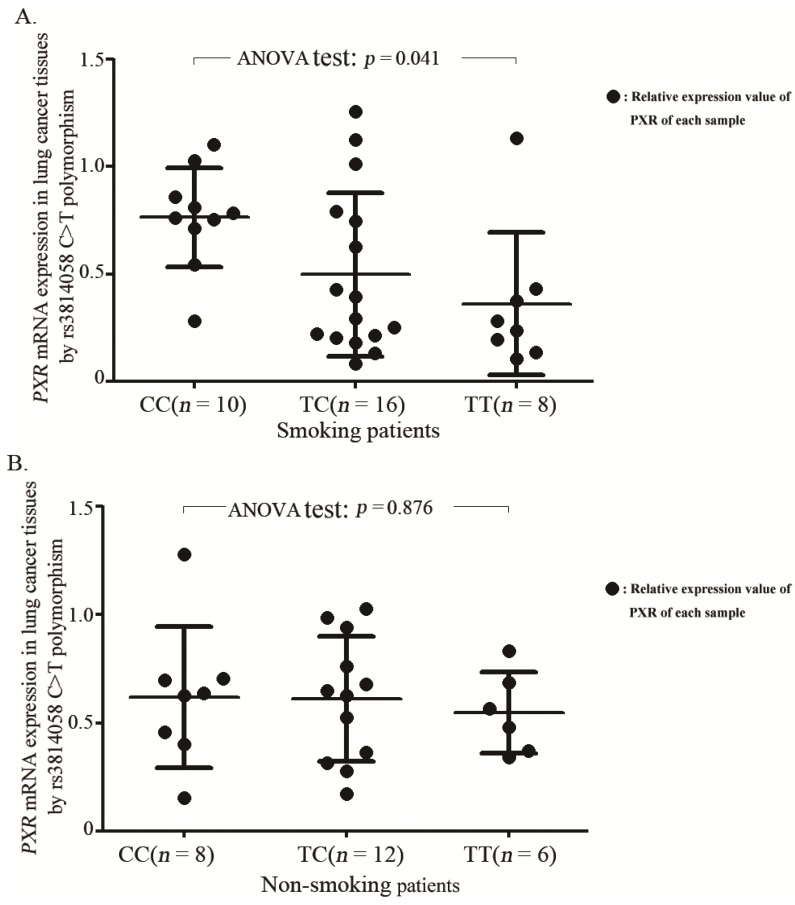
The difference of relative mRNA levels of *PXR* in lung cancer tissues by the rs3814058C>T genotypes with regard to smoking status. (**A**) In cancer tissues from smoking patients including current and former smokers; and (**B**) In cancer tissues from non-smoking patients; the differences of mRNA levels in tumor tissues with different genotypes were assessed by the One-way ANOVA test.

No significant difference of *PXR* mRNA levels was found in lung cancer tissues with different rs3814058C>T genotypes as presented in Figure S1. However, while divided into subgroups by smoking status, we found that compared to the rs3814058CC genotype, the rs3814058T variants had significantly lower mRNA expression levels in tissues from smoking patients including current and former smokers (CC: 0.76 ± 0.23; TC: 0.49 ± 0.38; TT: 0.36 ± 0.33; ANOVA test: *p* = 0.041, Figure 1A, but not in tissues from non-smoking patients (*p* = 0.876, Figure 1B).

## 3. Discussion

In these two independent case-control studies of 1559 lung cancer cases and 1679 controls conducted in southern and eastern Chinese populations, we found that the rs3814058T variants of rs3814058C>T in the 3'-UTR of *PXR* were significantly associated with an increased lung cancer risk in smokers of Chinese. A significant interaction between the rs3814058T variants and smoking was also observed. Furthermore, this SNP was functional as that the rs3814058T variants significantly decreased expression of *PXR* in comparison to the rs3814058CC genotype in those smoking individuals. However, there was no significant deviation in frequency distributions between cases and controls for the genotypes of SNPs rs3814055C>T and rs3732360C>T. To the best of our knowledge, this is the first report on genetic variants in *PXR* and susceptibility of lung cancer.

Evidences indicate that as a ligand-dependent transcription factor of the nuclear hormone receptor superfamily, PXR has a multitude of functions including toxic xenobiotics and endogenous metabolites metabolism, gut mucosal defense and energy homeostasis [27]. With its predominantly regulatory role in xenobiotic clearance via induction of metabolizing enzymes and drug transporters, *PXR* has been reported to be involved in the development of various cancers, such as breast cancer and colon cancer [28,29]. Although the direct role of PXR on lung tumorgenesis was unclear, it is well-established that PXR regulates the activity of the *CYP* gene family, which plays pivotal roles in metabolic transcription of tobacco-derived carcinogens and inhibits lung carcinogenesis [14,15,16,17]. Also, Low expression of *PXR* was observed in lung cancer cells [25]. All these suggested that PXR participates in the development of lung cancer. Recently, the associations between SNPs in the *PXR* gene and human diseases are extensively evaluated [6,7,8,9]. In the current study, based on a large sample size of a two-stage case-control study in Chinese population, we observed a novel association between the SNP rs3814058C>T in the 3'-UTR of *PXR* and lung cancer risk in smokers of Chinese, suggesting that the variants in *PXR* may be a valuable biomarker to predict risk of lung cancer.

In the stratification analysis, we found that the detrimental role of rs3814058C>T in lung cancer risk was more memorable in the subgroup of current and former smokers, and the rs3814058T variants also had a significant interaction with smoking on cancer risk. It is well known that tobacco consumption is the major risk factor of lung cancer [2]. Tobacco substrates can also stimulate PXR activities [16]. Moreover, results from our bioassay showed the SNP rs3814058C>T could significantly affect *PXR* mRNA expression in lung cancer tissues derived from smokers. These results indicated that the rs3814058C>T SNP-induced low *PXR* expression may cause more adverse effect in response to smoking stimulation and thus interacted with smoking on lung carcinogenesis.

To investigate the possible molecular mechanism on how the SNP rs3814058C>T influence the *PXR* expression, we performed bioinformatic analysis using the FuncPred tool from the SNPinfo Web Server (http://snpinfo.niehs.nih.gov/) [30]. The result showed that the C to T transposition of rs3814058C>T would result in a novel binding site of hsa-miR-501-5p. Therefore, it is biologically possible that rs3814058T variants significantly decrease expression of *PXR* via hsa-miR-501-5p regulation, which in turn weakens the detoxification of the carcinogens and thus facilitates tumorigenesis. However, further functional experiments to elucidate this biological mechanism of this SNP are obligatory.

Since our studies were two hospital-based case-control studies, restricted with Chinese Han populations, it is difficult to avoid selection bias and information bias. However, we have achieved an 83.0% study power (two-sided test, α = 0.05) to detect an OR of 1.25 for the rs3814058T variants (which occurred at a frequency of 67.2% in the controls) on lung cancer risk. The functional assay also supported the association. Therefore, it appears that our finding that the association between the *PXR* variant and lung cancer risk is unlikely to be achieved by chance. 

## 4. Experimental Section

### 4.1. Study Subjects

In the current study, two independent case–control studies were performed in southern and eastern Chinese populations. The sample collection and definition of studied variables, such as smoking, had been previously described [31,32,33]. Here in brief, a southern Chinese population was used as a discovery set with 1056 histopathologically confirmed primary lung cancer cases and 1056 age (±5 years) and sex-frequency matched cancer-free controls that were recruited from Guangzhou (China) and surrounding regions, and an eastern Chinese population was used as a validation set, which included 503 patients and 623 age (±5 years) and sex-frequency matched controls who were enrolled from Suzhou (China) and surrounding regions. The studies were approved by the institutional review boards of Guangzhou Medical University (Guangzhou, China) and Soochow University (Suzhou, China).

### 4.2. Single Nucleotide Polymorphism (SNP) Selection and Genotyping

SNPs located in the predicted 3000 bp promoter region, 5'-UTR, coding region and 3'-UTR of *PXR* are predicted to be putatively functional. Based on the data of Chinese population of HapMap database (http://hapmap.ncbi.nlm.nih.gov/) [34], we found that there were seven common SNPs with minor allele frequency (MAF) >5% in Chinese, among which the promoter SNPs and 3'-UTR SNPs were in completely linkage disequilibrium (LD) with each other, respectively, as shown in Figure S2A, B. Thus, we used the Haploview software 4.2 (Daly Lab at the Broad Institute, Cambridge, MA, USA) to select the tagger SNPs (TagSNPs) that could cover the genetic information of these 7 SNPs. Such three SNPs were selected in the current study that are rs3814055C>T in 5'-UTR, rs3732360C>T and rs3814058C>T in 3'-UTR of *PXR*.

Genomic DNA was extracted from 2 mL peripheral blood using the DNA Blood Mini Kit (Qiagen, Valencia, CA, USA). We genotyped the three tagSNPs using the TaqMan allelic discrimination Assay on an ABI7900 system (Applied Biosystems, Foster City, CA, USA) with the primes and probes as listed in Table S2. We further randomly selected 10% samples for each of the three SNPs to perform repeat assays, and the results were 100% concordant (Figure S2C).

### 4.3. PXR mRNA Expression Analysis

Because only the SNP rs3814058C>T of *PXR* was found to be significantly associated with lung cancer risk, we then determined whether the polymorphism had an effect on *PXR* gene expression by using the qRT-PCR method as previously described [35]. Total RNA of sixty lung cancer tissues were extracted by using the Trizol Reagent (Invitrogen, Carlsbad, CA, USA) and then reversely transcribed to complementary DNA by using the oligoT primer and the SuperscriptII (Invitrogen). mRNA expression levels of *PXR* and an internal reference gene *β-actin* were detected on the ABI Prism 7900 sequence detection system (Applied Biosystems) based on the SYBR-Green method. The primers for *PXR* were: 5'-GTTCAATGCGGAGACTGG-3' (forward) and 5'-GGGAGAAGAGGGAGATGG-3' (reverse) and for *β-actin* were: 5'-GGCGGCACCACCATGTACCCT-3' and 5'-AGGGGCCGGACTCGTCATACT-3'. Relative quantification of *PXR* mRNA was calculated according to the 2^−Δ*C*t^ method [36]. All analyses were performed in a blinded fashion with the laboratory persons unaware of genotyping data and each assay was done in triplicate.

### 4.4. Statistical Analysis

The Hardy-Weinberg equilibrium (HWE) was tested by a goodness-of-fit chi-square test to compare the expected genotype frequencies with observed genotype frequencies in cancer-free controls. The chi-square test was used to assess differences in the frequency distributions of demographic characteristics and genotypes of *PXR* SNPs between cases and controls. The association between each SNP and lung cancer risk was estimated using an unconditional logistic regression model with adjustment for age, sex, smoking status, drinking status and family history of cancer. A multiplicative interaction model was suggested to evaluate possible gene-environment interactions [37]. The differences of *PXR* mRNA levels in tumor tissues with different genotypes were assessed by the One-way ANOVA test. Moreover, the statistical power was calculated by using the PS Software (illiam D. Dupont and Walton D. Plummer, Nashville, TN, USA). All tests were two-sided by using the SAS software (version 9.3; SAS Institute, Cary, NC, USA) and *p* < 0.05 was considered to be statistically significant.

## 5. Conclusions

In conclusion, our data suggest that the SNP rs3814058C>T in the *PXR* gene is associated with an increased risk of lung cancer in Chinese smokers. The SNP rs3814058C>T of *PXR* may be a genetic biomarker for susceptibility to lung cancer. Validations with larger population-based studies in different ethnic groups and further biological assays are warranted to confirm our findings.

## Supplementary Materials

Supplementary materials can be found at http: http://www.mdpi.com/1422-0067/15/10/17457/s1.

## Supplementary Files

Supplementary File 1
