# Supplementary material for: A Functional Polymorphism in the 3'-UTR of PXR Interacts with Smoking to Increase Lung Cancer Risk in Southern and Eastern Chinese Smoker
Source: Int J Mol Sci. 2014 Sep 29;15(10):17457–68. doi: 10.3390/ijms151017457 (PMC4227172; doi:10.3390/ijms151017457)
Supplement: Supplementary File 1 [file ijms-15-17457-s001.pdf]

Supplementary Information

Table S1. Frequency distributions of selected variables in lung cancer patients and cancer-free controls.

Variables	Discovery Set (Southern Chinese)			Validation Set (Eastern Chinese)			Merged Set		
	Case (n = 1056) n (%)	Control (n = 1056) n (%)	p^a	Case (n = 503) n (%)	Control (n = 623) n (%)	p^a	Case (n = 1559) n (%)	Control (n = 1679) n (%)	p^a
Age (years)									
≤ 60	536 (50.8)	534 (50.6)	0.931	273 (54.3)	343 (55.1)	0.793	809 (51.9)	877 (52.2)	0.846
> 60	520 (49.2)	522 (49.4)		230 (45.7)	280 (44.9)		750 (48.1)	802 (47.8)	
Sex									
Male	746 (70.6)	746 (70.6)	1.000	345 (66.6)	433 (70.4)	0.496	1091 (70.0)	1185 (70.5)	0.710
Female	310 (29.4)	310 (29.4)		158 (31.4)	184 (29.6)		468 (30.0)	494 (29.5)	
Family history of cancer									
Yes	104 (9.9)	103 (9.8)	0.942	25 (5.0)	44 (7.1)	0.046	129 (8.3)	147 (8.8)	0.625
No	952 (90.1)	953 (90.2)		478 (95.0)	579 (92.9)		1430 (91.7)	1532 (91.2)	
Family history of lung cancer									
Yes	42 (4.0)	30 (2.8)	0.150	10 (2.0)	13 (2.1)	0.907	52 (3.3)	43 (2.6)	0.192
No	1014 (96.0)	1026 (97.2)		493 (98.0)	610 (97.9)		1507 (96.7)	1636 (97.4)	
Smoking status									
Ever	601 (56.6)	542 (51.4)	0.028	223 (44.4)	225 (35.8)	1.79×10^{-7}	824 (52.8)	765 (45.6)	1.48×10^{-6}
Never	455 (43.1)	514 (48.7)		280 (55.6)	400 (64.2)		735 (47.2)	914 (54.4)	
Drinking status									
Ever	229 (21.7)	227 (21.5)	0.042	64 (12.8)	115 (18.4)	0.017	293 (19.8)	342 (20.3)	0.049
Never	827 (78.3)	829 (78.5)		439 (87.2)	508 (81.6)		1266 (81.2)	1337 (79.7)	
Histological types									
Adenocarcinoma	384 (36.4)			231 (45.9)			615 (39.4)		
Squamous cell carcinoma	369 (34.9)			158 (31.4)			527 (33.8)		
Large cell carcinoma	43 (4.1)			23 (4.6)			66 (4.2)		
Small cell lung cancer	128 (12.1)			65 (12.9)			193 (12.4)		
Other carcinomas [b]	132 (12.5)			26 (5.2)			158 (10.2)		

Table S1. *Cont.*

Variables	Discovery Set (Southern Chinese)			Validation Set (Eastern Chinese)			Merged Set		
	**Case (*n* = 1056) *n* (%)**	**Control (*n* = 1056) *n* (%)**	***p*** [a]	**Case (*n* = 503) *n* (%)**	**Control (*n* = 623) *n* (%)**	***p*** [a]	**Case (*n* = 1559) *n* (%)**	**Control (*n* = 1679) *n* (%)**	***p*** [a]
Stages									
I	154 (14.6)			46 (9.2)			200 (12.8)		
II	94 (8.9)			53 (10.5)			147 (9.5)		
III	333 (31.5)			157 (31.2)			490 (31.4)		
IV	475 (45.0)			247 (49.1)			722 (46.3)		

[a] *p* values for a χ^2 test; and [b] Mixed-cell or undifferentiated carcinoma.

Table S2. Primary information on the TaqMan assay of three tagSNPs in the *PXR* gene.

SNP, rs no.	Variants	Primers	Probes [a]	Color (genotype)
rs3814055	C>T	CCTGAAGACAACTGTGGTCATTTT (forward)<br>CACGATTGAGCAAACAGGTAGAA (reverse)	FAM-AATCCCAGGTT***C***TC<br>HEX-AATCCCAGGTT***T***TC	Blue (CC)<br>Red (TT)<br>Green(TC)
rs3732360	C>T	GGGCTCCAGGCCTGTACTC (forward)<br>GGCCTCCTGGCTTCTCATCT (reverse)	FAM-CGGCAGG***C***GCATG<br>HEX-TCGGCAGG***T***GCAT	Blue (CC)<br>Red (TT)<br>Green(TC)
rs3814058	C>T	GGCATTCCACACCTAAGAACTAGTTT (forward)<br>CCCCTCTAGCCAAAAGTACATTATTT (reverse)	FAM-ATTTG***C***CATTAAACC<br>HEX-ATTTG***T***CATTAAACC	Blue (CC)<br>Red (TT)<br>Green(TC)

[a] Bold italic nucleotides indicate the polymorphic sites in probes.

Figure S1. The difference of relative mRNA levels of *PXR* in lung cancer tissues by the rs3814058C>T genotypes.

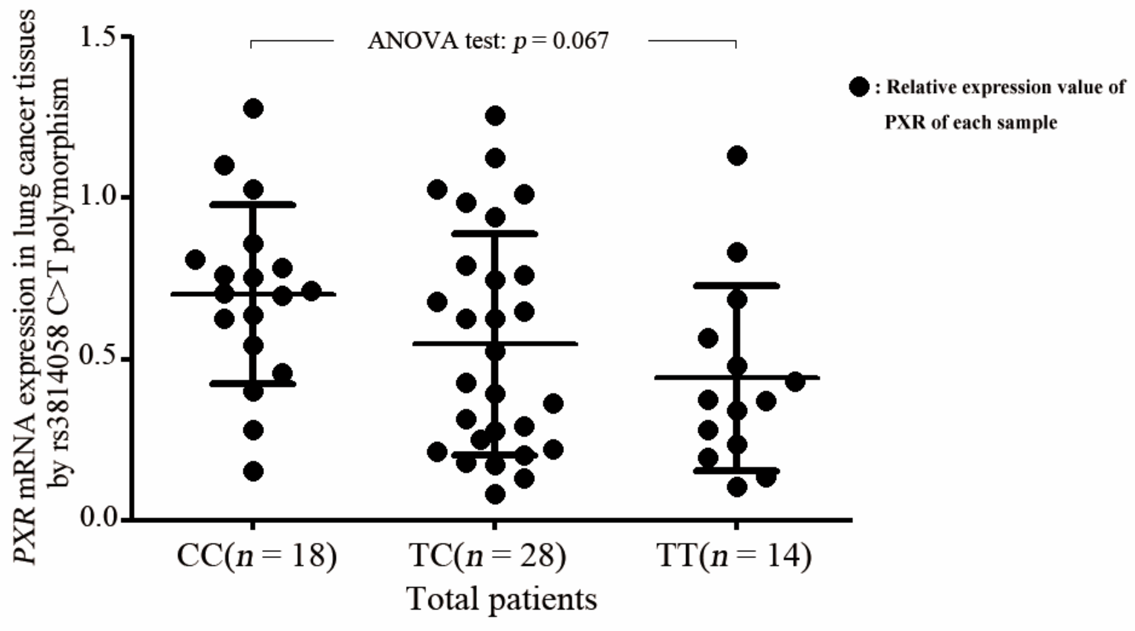

Figure S2. The gene structure of *PXR* and LD plot (**A**,**B**), and (**C**) genotyping production sketch of *PXR* SNPs.

PXR: 3q12-q13.3

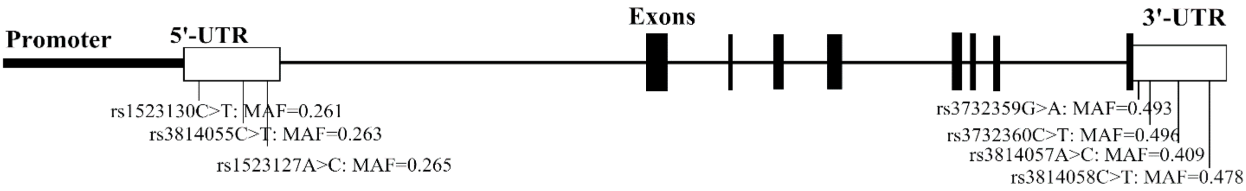

(**A**)

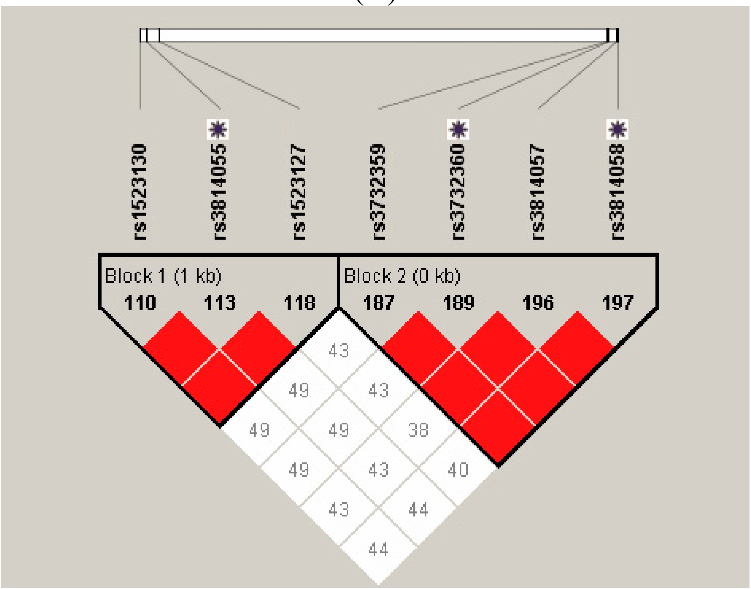

(**B**)

Figure S2. *Cont.*

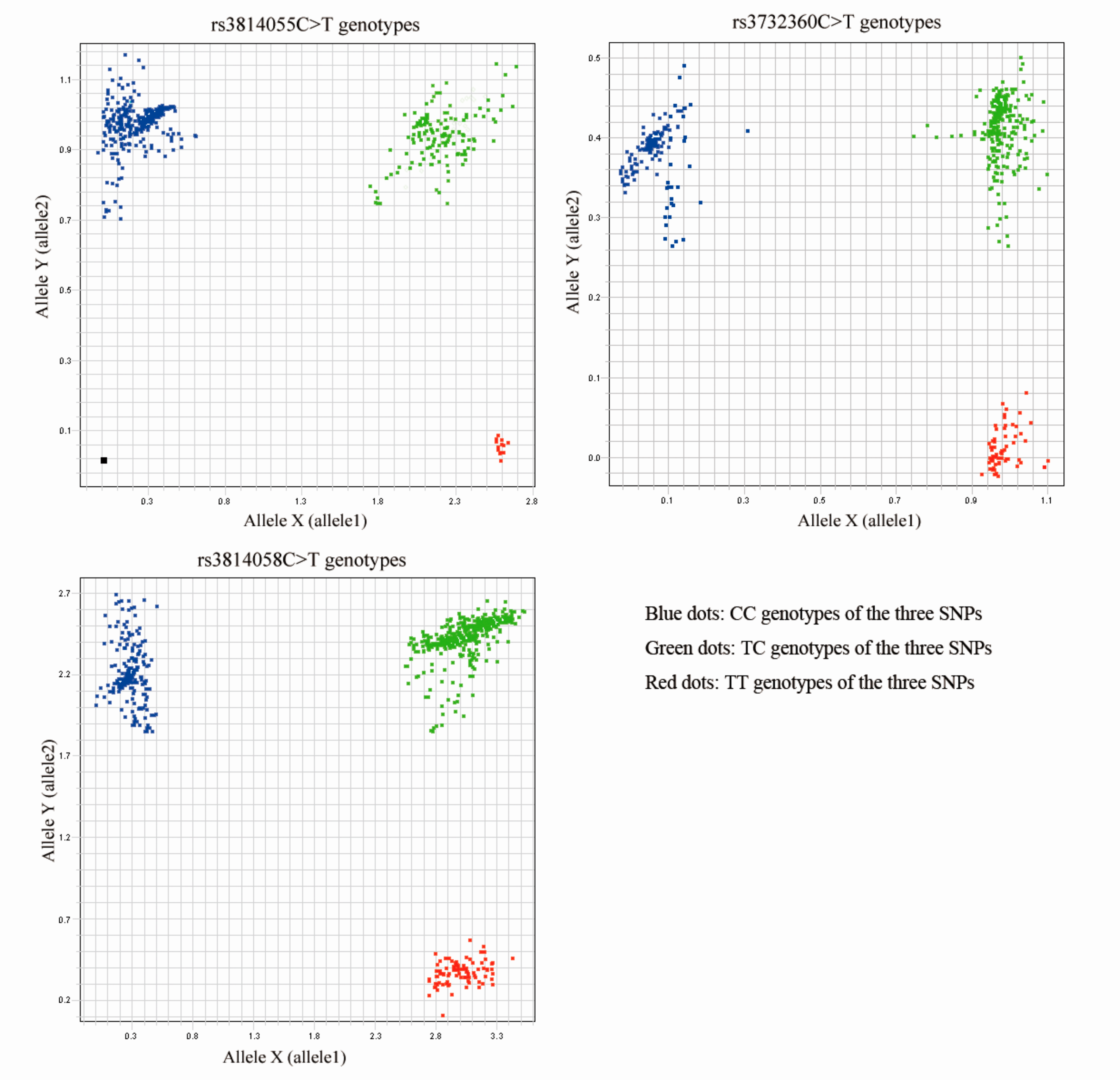

(**C**)
